# Evaluating the impact of electrode planes on regional lung function assessment

**DOI:** 10.3389/fphys.2024.1464377

**Published:** 2024-10-21

**Authors:** Ling Sang, Yutao Sun, Yu Lu, Zhimin Lin

**Affiliations:** ^1^ State Key Laboratory of Respiratory Diseases, Department of Critical Care Medicine, Guangzhou Institute of Respiratory Health, The First Affiliated Hospital of Guangzhou Medical University, Guangzhou Medical University, Guangzhou, China; ^2^ Guangzhou Laboratory, Guangzhou, China; ^3^ MidasMED Biomedical Technology, Suzhou, China

**Keywords:** electrical impedance tomography, electrode plane, regional lung function, forced vital capacity, spatial and temporal distribution

## Abstract

**Objective:**

The aim of the study was to explore the influence of the measurement plane on regional lung function assessed via electrical impedance tomography (EIT).

**Methods:**

The forced vital capacity (FVC) maneuver was prospectively performed in 30 healthy male volunteers. Simultaneously, EIT measurements were conducted at the 3rd, 4th, and 5th intercostal spaces (ICS). The EIT-based spirometry parameters are calculated in a similar manner to their original definitions. The spatial and temporal distributions of the corresponding functional images were assessed and compared among the measurement planes.

**Results:**

All subjects but one were able to perform the FVC maneuver according to the guidelines. Significant differences were found in 67% (6 out of 9) of the EIT-based parameters assessing the spatial and temporal distribution. The fEIT images were most homogeneous at ICS 4 compared to the other two measurement planes, except for the time required for 75% of FVC. The fEIT image FVC_EIT_ distributed toward dorsal regions when the measurement planes moved from ICS 3 to ICS 5, whereas the identified lung areas became smaller.

**Conclusion:**

The spatial and temporal distribution of the regional lung function measured via EIT was influenced by the measurement planes. We recommend adhering to the same measurement plane for before–after comparison. ICS 4 was recommended for the sitting subjects performing lung function testing.

## Introduction

Patients with various diseases require pulmonary function testing (PFT). For common obstructive lung diseases such as chronic obstructive pulmonary disease (COPD) and asthma, the forced vital capacity (FVC) maneuver is often used for diagnosis and monitoring (Halpin et al., 2021). Spatial non-uniformity is observed in bronchial abnormalities, and regional information could help characterize disease progression. Unfortunately, regional lung function cannot be assessed via spirometry (Gallardo Estrella et al., 2017). Electrical impedance tomography (EIT) is a novel functional radiation-free imaging technique (Frerichs et al., 2017). It measures the regional ventilation distribution over time by calculating the impedance changes in the corresponding areas. Previous studies showed that EIT can be used to measure the regional lung function during the FVC maneuver in patients with obstructive lung disease (Vogt et al., 2016; Vogt et al., 2018; Vogt et al., 2019).

During EIT measurement, 16 or 32 electrodes are attached around the thorax, which forms a so-called measurement (or electrode) plane. Insensible alternating currents are injected into the thorax, and the corresponding voltages are measured. Because the current path does not follow a straight line as an X-ray, air content changes in the measurement plane contribute most of the impedance changes measured by EIT, but off-plane effects also contribute to the EIT images. The location of the measurement plane impacts the volume–impedance ratio and may lead to misinterpretation of the results (Krueger-Ziolek et al., 2015; Karsten et al., 2016; Zhao et al., 2022). Until today, no one has investigated whether a similar regional lung function would be obtained at various measurement planes during EIT measurements.

This study aims to explore the influence of the measurement plane on regional lung function assessed via EIT.

## Methods

The prospective observational study was approved by the Institutional Research and Ethics Committee of the Guangzhou Medical University (2022-161). Informed consent was obtained from all subjects prior to conduction of the study. A total of 30 lung healthy volunteers were prospectively examined via EIT (male: female, 30:0; age, 25.9 ± 3.5 years; height, 177.6 ± 5.0 cm; and weight, 75.9 ± 10.7 kg).

**FIGURE 1:**
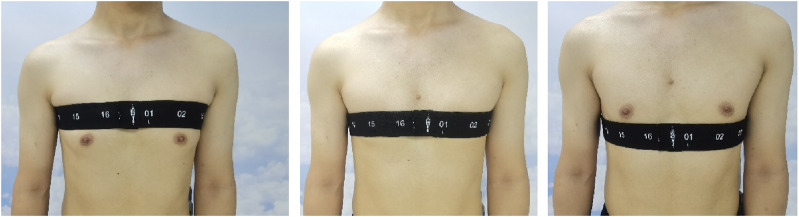
Illustration of the measurement plane positions. Left, third intercostal spaces; middle, fourth intercostal spaces; and right, fifth intercostal spaces. The numbers on the electrode belt marked the position of the electrodes.

The subjects were spontaneously breathing in the sitting position. A belt with 16 equidistantly fixed electrodes was placed around the chest in one transverse plane (e.g., the level of the third intercostal spaces (ICS) at the parasternal line). The FVC maneuver was conducted at least 3 times according to the ATS 2019 standard (Graham et al., 2019). The variation of the forced expiratory volume at 1 s (FEV1) and FVC were within 0.15 L between the highest two efforts (confirmed using a spirometer, HI-101; CHEST M.I., INC., Tokyo, Japan). After a brief break (∼5 min), the electrode belt position altered from the third ICS to the fourth ICS and then to the fifth ICS (Figure 1). The FVC maneuver was repeated according to the ATS guidelines. To avoid the influence of the breast on the exact belt position, only male subjects were included in the study. Raw EIT data were acquired using VenTom-100 (MidasMED Biomedical technology, Suzhou, China) at a scan rate of 20 images/s using excitation currents of 1 mArms applied through opposite electrodes. Image reconstruction was accomplished by the GREIT algorithm (Adler et al., 2009). The baseline for image reconstruction was obtained individually for each subject during quiet tidal breathing before the FVC maneuver.

The EIT waveforms in each of image pixels were used for the analysis of regional lung function parameters. In every waveform, the beginning and the end of the forced full expiration were detected.

Thereafter, the following EIT-derived parameters in each EIT pixel were calculated:

(1) *FEV1*_*EIT*_, the difference between relative impedance changes (rel.ΔZ) at total lung capacity (TLC) and the forced expiration after 1 s.
(2) *FVC*_*EIT*_, the difference between rel.ΔZ at TLC and the end of forced expiration.
(3) *FEV1/FVC*_*EIT*_ ratio.
(4) The forced mid-expiratory flow (*FEF25%–75%*_*EIT*_), the average rel.ΔZ/time between 25% and 75% of FVC.
(5) *T75*, the time required for 75% of FVC.

The abovementioned EIT-derived parameters formed the corresponding functional EIT (fEIT) images. Based on the fEIT images, the global inhomogeneity (*GI*) index was calculated (Zhao et al., 2009) to assess the spatial non-uniformity of the regional lung function. The lung regions were identified as described previously (Zhao et al., 2010). The size of lung regions in pixel was denoted as *A*_*lung*_. Compared to the original GI index, the pixel-wise parameter values were used instead of tidal variation (Frerichs et al., 2021). In addition, we calculated a parameter similar to the center of ventilation (Frerichs et al., 1998) to assess the balance of the volume distribution for the FVC fEIT image (denoted as *CoV*_*FVC*_). When the pixel *FEV1/FVC*_*EIT*_ value was lower than 0.7, this pixel region was considered abnormal. The number of abnormal pixels over *A*_*lung*_ was denoted as *R*_*abnormal%*_.

### Statistical analysis

Data analyses were performed using MATLAB R2023a (the MathWorks Inc., Natick, United States). The Lilliefors test was used for normality testing. For normally distributed data, results were expressed as mean ± standard deviation. One-way ANOVA was used to compare the EIT-based parameters among different measurement planes. *p*-value <0.05 was considered statistically significant. For statistically significant parameters, paired *t*-test was used to further compare the differences between two measurement planes. Significance levels were corrected for multiple comparisons using Holm’s sequential Bonferroni method.

## Results

**FIGURE 2:**
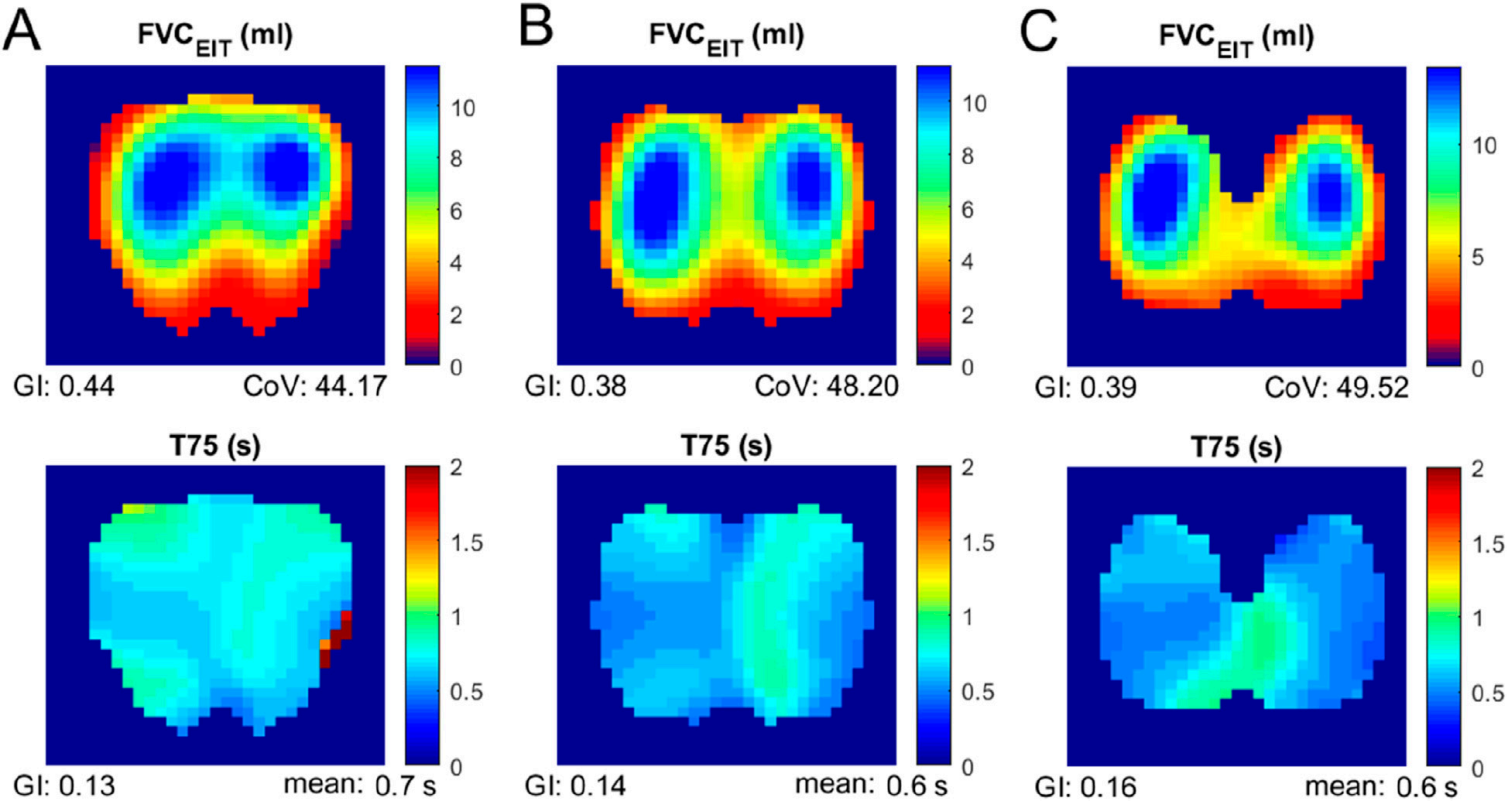
Functional EIT images of FVC (top) and T75 (bottom) measured at various electrode planes. Column **(A)**, third intercostal spaces; column **(B)**, fourth intercostal spaces; and column **(C)**, fifth intercostal spaces.

**FIGURE 3:**
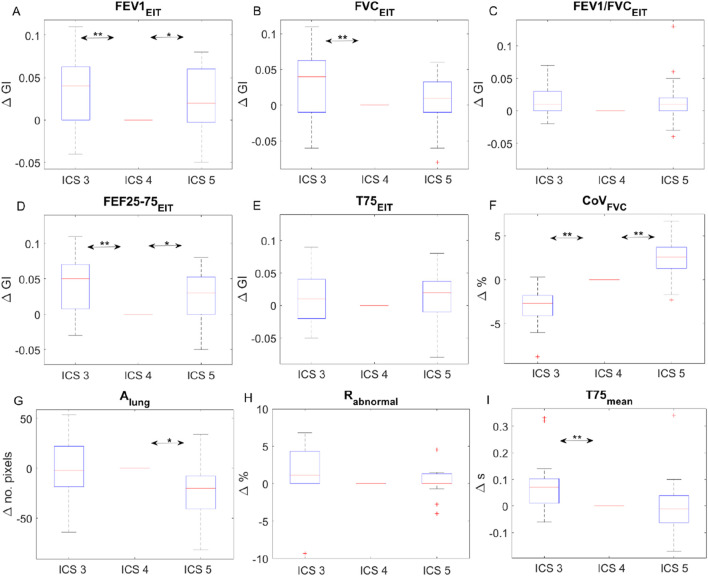
Individual differences of EIT-based parameters at intercostal spaces (ICS) 3 and 5 compared to that at ICS 4. The boxes mark the quartiles, while the whiskers extend from the box out to the most extreme data value within 1.59, the interquartile range of the sample. Change in GI of fEIT FEV1 **(A)**, FVC **(B)**, the ratio **(C)**, FEF25-75 **(D)**, and T75 **(E)**; change in CoV **(F)**, lung area **(G)**, abnormal region **(H)** and the average time of T75 **(I)**. Red pluses are the outliers. *P = 0.01; **P = 0.001.

All subjects but one were able to perform the FVC maneuver according to the ATS guidelines 2019. Therefore, data from 29 subjects were collected and analyzed. The typical fEIT of FVC and T75 of one subject is plotted in Figure 2. The shapes of the identified lung regions were different. The EIT-based parameters assessing the spatial and temporal distribution of the fEIT images are presented in Table 1. Significant differences were found in 67% (6 out of 9) of the evaluated parameters. The individual differences compared to that at ICS 4 are plotted in Figure 3. The fEIT images were most homogeneous at ICS 4 compared to other two measurement planes, except for *T75*_*EIT*_ (Figure 3E). The fEIT image FVC_EIT_ distributed toward dorsal regions when the measurement planes moved from ICS 3 to ICS 5 (*CoV*_*FVC*_, Figure 3F), whereas the identified lung areas became smaller (*A*_*lung*_, Figure 3G). The Rabnormal (Figure 3H) and T75 mean (Figure 3I) were higher in ICS 3.

**TABLE 1:** Comparison of EIT-based assessments at different measurement planes.

Parameter		ICS3	ICS4	ICS5	p
GI	*FEV1*	0.41 ± 0.04	0.38 ± 0.03	0.40 ± 0.03	<0.001 **
	*FVC*	0.41 ± 0.05	0.38 ± 0.04	0.39 ± 0.03	0.01 *
	*FEV1/FVC*	0.06 ± 0.05	0.04 ± 0.04	0.05 ± 0.03	0.77
	*FEF25%–75%*	0.42 ± 0.05	0.38 ± 0.04	0.41 ± 0.03	0.006 *
	*T75*	0.12 ± 0.05	0.12 ± 0.07	0.14 ± 0.07	0.41
*CoV*_*FVC*_ (%)		44.0 ± 2.1	47.0 ± 2.9	49.5 ± 3.2	<0.001 **
*A*_*lung*_ (no. of pixels)		449 ± 31	448 ± 33	426 ± 36	0.01 *
*R*_*abnormal%*_ (%)		1.50 ± 6.8	0.00 ± 0.70	0.00 ± 1.5	0.22
*T75*_*mean*_ (s)		0.76 ± 0.16	0.68 ± 0.09	0.67 ± 0.13	0.02 *

GI, global inhomogeneity index; FEV1, forced expiratory volume at 1 s; FVC, forced vital capacity; FEF, forced expiratory flow; T75, time required for 75% of FVC; CoV, center of ventilation; A_lung_, size of the lung areas in pixel; No, number; R_abnormal%_, the percentage of abnormal regions; ICS, intercostal spaces.

## Discussions

In the present study, we found that the spatial and temporal distribution of the regional lung function measured via EIT was influenced by the measurement planes. For before–after comparison, the same measurement plane should be used. ICS 4 was recommended for sitting subjects.

The use of EIT to assess lung function can be dated back to the 1990s (Sahalos et al., 1992; Eyüboğlu et al., 1995). Chest EIT is based on the assumption of a linear relationship between relative impedance changes and lung volume changes during inspiration and expiration. Since flow is calculated as the derivatives of volume, the derivatives of relative impedance are considered to be proportional to the inspiratory and expiratory flows. The EIT-based spirometry parameters are calculated in a similar manner to their original definitions, except that the regional impedance change and corresponding derivatives are used as substitutes for the volume and flow, respectively (Sang et al., 2020). The information of the regional lung function or flow limitation was proposed to monitor the disease progression [(Zhao et al., 2013; Lehmann et al., 2016)], assess the treatment effects [(Ma et al., 2022; Longhini et al., 2020)], or even guide the treatment program [(Li et al., 2023)]. To guarantee the monitoring purpose, Reifferscheid et al. explored the EIT data from the same subjects on different days and confirmed the reproducibility (Reifferscheid et al., 2011). They also found that ventilation distribution was different on two different measurement planes. Later, several studies systematically demonstrated the influence of measurement planes on the ventilation distribution (Krueger-Ziolek et al., 2015; Karsten et al., 2016; Zhao et al., 2022). We found that the regional lung function was also influenced by the measurement planes. However, unlike the investigation of influence on the volume distribution, we did not focus on the impedance–volume ratio. Instead, we analyzed the clinical parameters that reflect the lung function. One reason was that the subjects were all in an upright position, and we did not extend the measurement plane to lower than the ICS 5, which was still a recommended range (Frerichs et al., 2017). Nevertheless, the influence of the diaphragm is not neglectable. *CoV*_*FVC*_ showed a clear trend toward the dorsal region as the measurement plane moved toward the caudal direction (Figure 3F). At the same time, the lung size was also influenced (Figure 3G).

The distribution heterogeneity (GI) of fEIT images *FEV1*_*EIT*_, *FVC*_*EIT*_, and *FEF25–75%*_*EIT*_ achieved the lowest value (most homogeneous) at ICS 4 (Figures 3A–C). The GI calculation is influenced by the identified lung region (Yang et al., 2021a). Therefore, the change in *A*_*lung*_ might have influenced the GI values. In addition, the method we used in the present study to identify lung regions mirrors the lung regions left to right (Zhao et al., 2010). Any anatomical asymmetry (e.g., heart) will influence the GI values as well. Although statistical significances were found, the average differences of GI were smaller than 0.05 (Figures 3A–E). In a previous study, the variation of GI for tidal ventilation was explored (Yang et al., 2021b). A standard deviation of 0.04 was found in a group of 75 healthy volunteers. Hence, it is unclear whether the differences found in the present study were clinically significant. Similarly, the mean difference of T75 was smaller than 0.1 s, which might also be ignorable clinically (∼14% of the absolute value ∼0.7 s). The clinically significant levels of various lung diseases have to be confirmed in future studies.

For the abnormal regions *R*_*abnormal*_, some subjects exhibited 5% regions with flow limitations. Although the study subjects were all lung healthy volunteers with normal lung function assessed via spirometry, the EIT parameters might be more sensitive for early detection of the regional abnormal lung function. Another possible reason was that the lung region identification method mirroring left–right lungs might not be suitable for measurement plane ICS 3. It is worth noting that in the original article, the method was proposed for supine subjects and for the measurement plane of ICS 5.

The limitations of the present study are acknowledged. The study subjects were men. Due to the size of the breast, the exact placement of the electrode plane on the female subjects would be hard to control and might introduce bias. However, how measurement planes affect regional lung function in women is still unknown. The age of the tested subjects was between 20 and 35 years. Although the data quality of the FVC maneuver is in general poor in children and adolescents, the FVC decreases as the age increases in the elderly; whether the findings of the current study can be extended to younger or older subject groups is unknown.

## Conclusion

Measurement planes could influence the spatial and temporal distribution of the regional lung function measured via EIT. Although the differences were relatively small, the same measurement plane should be used for intra-subject comparison.

## Data Availability

The original contributions presented in the study are included in the article/Supplementary Material; further inquiries can be directed to the corresponding author.
